# 511 Gastrointestinal Acute Radiation Syndrome Delays Epithelialization of Thermal Burn Wounds in Swine

**DOI:** 10.1093/jbcr/iraf019.140

**Published:** 2025-04-01

**Authors:** Babita Parajuli, Anna Carpenter, Tim Horseman, Hengying Ellery, Joseph Anderson, David Burmeister

**Affiliations:** Uniformed Services University of the Health Sciences; AFRRI; Uniformed Services University of the Health Sciences; Uniformed Services University of the Health Sciences; Uniformed Services University of the Health Sciences; Uniformed Services University of the Health Sciences

## Abstract

**Introduction:**

The current geopolitical climate increases the likelihood of nuclear attacks which would cause complex injuries such as concomitant thermal burns and radiation exposure (i.e., combined injury). Combined injuries are not completely understood, and not much is known on how gastrointestinal acute radiation syndrome (GI-ARS) affects burn wound healing.

**Methods:**

We created six-5 x 5cm contact burns on the dorsum of two Sinclair minipigs, with one animal also receiving 8 Gy hemibody irradiation with a linear accelerator. Wounds were tattooed, and photographed, wet debrided, biopsied, and re-dressed on days 3, 7, 14, 21, and 28. Biopsies were used for histological and Western blot analyses.

**Results:**

The leukocyte count was consistently higher in the irradiated animal compared to the unirradiated animal, which at day 28 was 17.44 and 10.53 cells/μl, respectively. Burn wounds from both animals contracted at similar rates, which at day 28 reached 86.2 ± 2.4% and 79.5 ± 3.5% in the irradiated and unirradiated animals, respectively (P=0.722). This was also accompanied by a non-significant difference in SMA protein levels, which were slightly higher in wounds from the irradiated animal. Alternatively, irradiation significantly delayed reepithelization (P< 0.001) which was evident as early as day 14 where wounds from the unirradiated animal were 89.6 ± 10.4% reepithelialized compared to 12.9 ± 12.9% in the irradiated animal. This was further evidenced by blinded histopathologist scoring that revealed significantly higher ulceration (P=0.006), decreased fibroplasia (P=0.006) and epidermal hyperplasia (P=0.011) in wounds from the irradiated animal. Picrosirius red staining also revealed less organized collagen bundles in wounds from the irradiated animal at all timepoints. Western blotting also showed a trend to higher levels of GAPDH-normalized E-cadherin in irradiated wounds (0.36 ± 0.04) compared to unirradiated wounds (0.22 ± 0.02) at day 28 (P=0.08, n=4 wounds/group).

**Conclusions:**

Taken together, we show that GI-ARS negatively affects epithelialization (but not contraction) of thermal burns which may be due to an appropriate immune response. Future studies will further characterize the mechanistic basis for delayed wound healing after radiation exposure.

**Applicability of Research to Practice:**

This study establishes a large animal model that can be used to direct the treatment of cutaneous radiation injuries as well as thermal injuries during concomitant radiation exposure.

**Funding for the Study:**

Startup package funding

